# Insights From a Text Messaging–Based Sexual and Reproductive Health Information Program in Tanzania (m4RH): Retrospective Analysis

**DOI:** 10.2196/10190

**Published:** 2018-11-01

**Authors:** Patrick S Olsen, Kate F Plourde, Christine Lasway, Eric van Praag

**Affiliations:** 1 Health Services Research, Global Health, Population, and Nutrition FHI 360 Durham, NC United States; 2 Research Utilization, Global Health, Population, and Nutrition FHI 360 Durham, NC United States; 3 Palladium: Make It Possible Health Practice, Americas Washington, DC United States; 4 Public Health Consultant Dar es Salaam United Republic Of Tanzania

**Keywords:** data analysis, mobile phone, mHealth, short message service, user engagement

## Abstract

**Background:**

Many mobile health (mHealth) interventions have the potential to generate and store vast amounts of system-generated participant interaction data that could provide insight into user engagement, programmatic strengths, and areas that need improvement to maximize efficacy. However, despite the popularity of mHealth interventions, there is little documentation on how to use these data to monitor and improve programming or to evaluate impact.

**Objective:**

This study aimed to better understand how users of the Mobile for Reproductive Health (m4RH) mHealth intervention engaged with the program in Tanzania from September 2013 to August 2016.

**Methods:**

We conducted secondary data analysis of longitudinal data captured by system logs of participant interactions with the m4RH program from 127 districts in Tanzania from September 2013 to August 2016. Data cleaning and analysis was conducted using Stata 13. The data were examined for completeness and “correctness.” No missing data was imputed; respondents with missing or incorrect values were dropped from the analyses.

**Results:**

The total population for analysis included 3,673,702 queries among 409,768 unique visitors. New users represented roughly 11.15% (409,768/3,673,702) of all queries. Among all system queries for new users, 46.10% (188,904/409,768) users accessed the m4RH main menu. Among these users, 89.58% (169,218/188,904) accessed specific m4RH content on family planning, contraceptive methods, adolescent-specific and youth-specific information, and clinic locations after first accessing the m4RH main menu. The majority of these users (216,422/409,768, 52.82%) requested information on contraceptive methods; fewer users (23,236/409,768, 5.67%) requested information on clinic location. The conversion rate was highest during the first and second years of the program when nearly all users (11,246/11,470, 98.05%, and 33,551/34,830, 96.33%, respectively) who accessed m4RH continued on to query more specific content from the system. The rate of users that accessed m4RH and became active users declined slightly from 98.05% (11,246/11,470) in 2013 to 87.54% (56,696/64,765) in 2016. Overall, slightly more than one-third of all new users accessing m4RH sent queries at least once per month for 2 or more months, and 67.86% (278,088/409,768) of new and returning users requested information multiple times per month. Promotional periods were present for 15 of 36 months during the study period.

**Conclusions:**

The analysis of the rich data captured provides a useful framework with which to measure the degree and nature of user engagement utilizing routine system-generated data. It also contributes to knowledge of how users engage with text messaging (short message service)-based health promotion interventions and demonstrates how data generated on user interactions could inform improvements to the design and delivery of a service, thereby enhancing its effectiveness.

## Introduction

Mobile phones are a well-established platform for health education and behavior change, and as global levels of phone and internet access continue to rise, so does the application of mobile health (mHealth) approaches across multiple public health sectors [1-3]. Many mHealth interventions have the potential to generate and store vast amounts of system-generated data that could provide insight into user engagement, programmatic strengths, and areas that need improvement to maximize efficacy. However, despite the popularity of mHealth interventions, there is little documentation on how to use these data to monitor and improve programming or to evaluate impact.

The utilization of mHealth program data for process evaluation creates an opportunity to complement conventional impact evaluation approaches. The analysis of mobile phone program use data allows researchers to examine information from all participants compared with traditional process evaluation approaches (such as in-depth interviews or focus group discussions and questionnaires) that collect information from a subset of program participants. It is more cost effective than other methods, and because analytics are collected in real-time, the approach improves efficiency and eliminates the potential for recall or interview bias [4,5]. Research demonstrates that the analysis of these data does provide valuable information for program evaluation. For example, the examination of user experience data, including the number of menus accessed, from an mHealth intervention delivered to young and middle-aged male alcohol users in Scotland allowed researchers to measure the fidelity of the intervention delivery, user engagement, and message comprehensibility—all critical indicators of programmatic success [4].

As summarized by Perski et al, several conceptual models have been presented in the literature to depict the behavior change theory underlying digital behavior change interventions [6]. Across these, the level and quality of user engagement with digital health interventions are considered key factors in the intervention’s effectiveness [6-8]. The literature presents a broad definition of user engagement that often encompasses the number of intervention interactions, the relevance and relatability of the content, and repeat use [9,10]. User engagement is influenced by multiple aspects of intervention design such as resonance of messages, message delivery pattern, and program interactivity [11].

To better understand how users of the Mobile for Reproductive Health (m4RH) mHealth intervention engaged with the program, we conducted a retrospective analysis of m4RH system use data from 1 country (Tanzania) from September 2013 to August 2016. m4RH is a short message service (SMS) text messaging-based sexual and reproductive health (SRH) information program. It was initially developed in 2009 to provide information about the full range of contraceptive methods available locally and was first implemented in Kenya and Tanzania. The program has since been adapted in multiple countries, including Uganda and Rwanda, and its content has been expanded to include additional SRH topics such as HIV, sexually transmitted infections (STIs), and puberty.

User engagement can be measured using both subjective (qualitative) and objective (quantitative) measures [6,10]. Previously published research describes the results of qualitative data collection efforts to understand the acceptability and quality of interactions with the m4RH system [12,13]. This publication focuses on the retrospective analysis of subjective measures of engagement: system use metrics. The concept of user engagement, for the purposes of this analysis, builds upon the work of previous authors and is characterized by the following attributes as defined in Table 1 [6,14].

**Table 1:** Definitions pertaining to user engagement attributes.

Dimensions	Indicator	Definition
Environment [15]: associated with factors thought to influence engagement such as access, social norms, and time use patterns	Location	Sum of unique requests for all wards per district
Interaction [6]: how often users interact with the system and over what period; a key dimension of engagement	New users	The number of unique users that accessed the system from September 2013 to August 2016
	Return users	The number of unique users that accessed the system at least once per month for 2 or more months
	Repeat users	The number of unique users that accessed the system more than once per month (does not include users who accessed the same menu items twice during the same month)
	Acquisition	Users (return and new) that accessed the system each month
Depth [6,8]: level of content consumed; a key dimension of engagement	Activation	Percent users who requested a submenu after receiving the main menu (indicates navigation through m4RH main menus)
	Active use	Percent of activated users who request content keywords after receiving submenu prompt
Loyalty [6,8,16]: Degree of involvement over time, retention—“stickiness”; a key dimension of engagement	Conversion	Percent of users who become active users (ie, request content)
	Churn	Percent users lost (uses that do not become active users)

m4RH is an on-demand system that requires users to navigate and request information before accessing content. Users can access content in 2 ways: by accessing the navigation menu, which takes an average of 3 steps to access content or directly entering a content code after the welcome menu (ie, 2 steps). In Tanzania, participants access the m4RH program by SMS text messaging “m4RH” to short code “15014” to receive a menu of choices for accessing information on a variety of SRH topics including contraceptive methods, family planning (FP) clinic locations, role model stories (story installments modeling positive health attitudes, norms, and behaviors), and so forth. To access any of the menu options, respondents reply with another SMS text message using a numerical code corresponding to each menu item. Respondents only receive SMS text messages as they request them; no follow-up or reminder SMS text messages are sent after the initial query. To encourage respondent participation, respondents incur no cost for either sending or receiving SMS text messages when interacting with the m4RH system.

## Methods

### Data Analysis

Secondary data analysis was conducted using longitudinal data collected by mobile SMS text messaging from respondents of the m4RH program from 127 districts in Tanzania from September 2013 to August 2016. Participant interactions were extracted from system logs that captured each query to the m4RH system, including respondent mobile phone number (used to identify unique system interactions), information requested (eg, contraceptive methods, HIV and STI prevention, sex and pregnancy, and puberty), and date and time of the query. The analysis utilized descriptive analysis (proportions and means) of participant interactions and adapted the conversion funnel framework and indicators (mainly used in e-commerce) to map out and assess the journey of a user in a Web-based system. Conversion funnels are grounded in consumer decision-making theory and lifecycle, which mirrors behavior change models such as the transtheoretical model [17].

This study was reviewed and approved by the federally registered institutional review board of FHI 360, the Protection of Human Subjects Committee, and the National Institute for Medical Research Tanzania Institutional Review Board. Respondents did not receive reimbursement for participating in any study activity.

### Data Cleaning

From September 2013 to August 2016, there were 4,112,460 system queries. Among those queries, 35,978 were invalid menu selections or text, 402,780 were duplicated menu selections sent in succession, and 5663 were m4RH specified incorrectly (ie, mr4h, m4hr, mrh4, or mfrh). After removing invalid and duplicate queries, the total population for analysis included 3,673,702 queries among 409,768 unique visitors.

Data cleaning and analysis was conducted using Stata 13 (Stata Corp, College Station, TX, USA). For analysis, all data were combined into 1 dataset consisting of multiple data points per user. This repeated measures dataset was sorted by users’ mobile number (unique ID) and date of system query. In this analysis, the proportions and frequencies of respondent queries are aggregated yearly unless specified, for ease of interpretability in user engagement over time. The data were examined for completeness (queries with missing time or date) and “correctness” (invalid menu option, duplicated menu selections sent in succession, and incorrectly specifying the program name, ie, mr4h, m4hr, mrh4, or mfrh). In addition, data were examined to identify illegitimate or implausible entries, for example, users sending duplicate queries in succession. Implausible entries identified during this process were excluded from analysis. No missing data were imputed. Respondents with missing or incorrect values were dropped from the analyses. To date, m4RH has reached 409,768 unique users in Tanzania living in 127 of the country’s 129 districts.

## Results

### Environment

The environment is hypothesized to influence engagement as social norms about seeking health information and mobile phone use, access to digital technology, and time use patterns are often similar in like settings [6]. Setting or location is one dimension of context. Through this analysis, we were able to examine the location using clinic location as a proxy. Only 5.67% (23,236/409,768) of new users requested information on the location of nearby clinics, and among these users, 33.30% (7,737/23,236) requested information on clinics within a given district. The region with the most requests was Dar es Salaam, representing 50.91% (3939/7737) of all requests, followed by Dodoma, Mwanza, and Arusha, representing 9.65% (747/7737), 8.52% (659/7737), and 7.60% (588/7737), respectively (see Table 2). These are all urban districts.

### Interaction

During the analysis period, 409,768 new users initiated m4RH in Tanzania. New users represented roughly 11.15% (409,768/3,673,702) of all queries. Table 3 shows total interactions for new and return users. Across all time points, on average 381 new users accessed the system per day and interacted with the system 5 times within 24 hours and 7 times per month. Among new, return, and repeat queries, the average duration between the first and last daily query was 64 minutes, and the duration between each query was 17 minutes.

**Figure 1:**
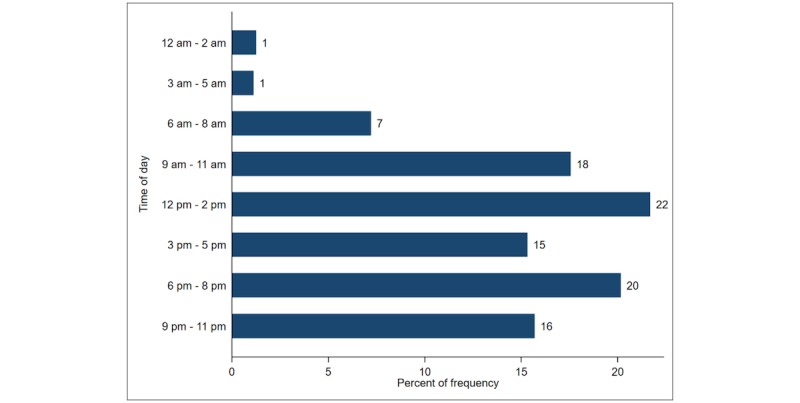
Overall user access time in 3-hour increments.

**Figure 2:**
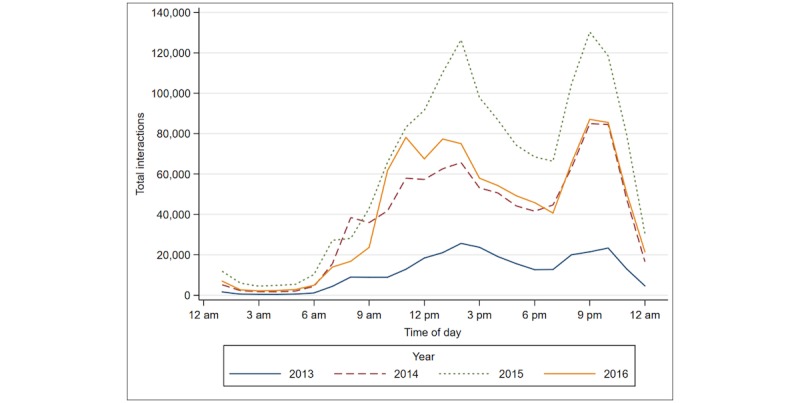
User access time, hourly by year.

Most daily queries occurred between 12 pm and 2 pm and between 6 pm and 8 pm (796,678/3,673,702, 21.69%, and 740,682/3,673,702, 20.16%, respectively). The fewest queries took place between 12 am and 5 am (see Figure 1). Access time shows similar trends across years (Figure 2).

**Table 2:** Frequency of wards requested per district in Tanzania from September 2013 to August 2016.

District	Value (N=7737), n (%)
Dar es Salaam	3939 (50.91)
Dodoma	747 (9.65)
Mwanza	659 (8.52)
Arusha	588 (7.60)
Mbeya	302 (3.90)
Kigoma	261 (3.37)
Tanga	193 (2.49)
Njombe	189 (2.44)
Iringa	174 (2.25)
Geita	170 (2.20)
Morogoro	158 (2.04)
Pwani	139 (1.80)
Simiyu	84 (1.09)
Ruvuma	68 (0.88)
Singida	52 (0.67)
Mjini Magharibi	13 (0.17)
Mara	1 (0.01)

**Table 3:** Total interactions by new and return users.

Users	Year, n (%)					Total (N=3,673,702), n (%)
	2013 (N=280,304)	2014 (N=923,661)	2015 (N=1,475,428)	2016 (N=994,309)		
New users	43,746 (15.61)	116,654 (12.63)	151,903 (10.30)	97,465 (9.80)	409,768 (11.15)	
Return users	4731 (1.69)	36,887 (4.00)	70,703 (4.80)	38,594 (3.88)	150,915 (4.11)	

### Depth

After first accessing the m4RH main menu, 89.58% (169,218/188,904) of users requested FP, contraceptive methods, youth-specific information, role model stories, or clinic locations submenus. The majority of all users (216,422/409,768, 52.82%) requested the contraceptive methods submenu. Among those that requested the submenu, 24.15% (98,969/409,768) requested additional information on natural FP methods and only 9.02% (36,968/409,768) requested information on intrauterine contraceptive devices (IUCDs).Close to the same number of users accessed FP and role model stories.

The types of information requested on contraceptive methods over the 36-month period followed similar trends relative to the number of new users requesting contraceptive method information each year. Few users requested information on clinic location (see Table 4).

As shown in Table 5, across all years, users were most interested in FP methods. Within the pregnancy prevention menu, roughly one-quarter (98,969/409,768, 24.15%) of all queries were requests for information on natural FP, as shown in Table 6 below.

Queries for information on other pregnancy prevention methods were more equally distributed, ranging from roughly 10% to 17%. The pregnancy prevention method with the fewest queries was IUCD, representing only 9.02% (36,968/409,768) of all pregnancy prevention queries (Table 6). Among respondents seeking information on youth content (Table 7), the most commonly requested topic was About Sex (34,542/249,368, 13.85%), while requests for Puberty and Choices represented similar proportions, at 6.43% (16,043/249,368) and 5.31% (13,238/249,368), respectively.

Menu content for “General Family Planning” and “Youth Content” was not implemented in Tanzania until 2015 and can help explain the lower frequencies among the 2 menu options for 2014 and 2015.

On average, users queried the system 5.56 times per month on the m4RH content described above. The rate of users that accessed m4RH and become active users declined slightly from to 98.05% (11,246/11470) in 2013 to 87.54% (56,969/97,465) in 2016.

**Table 4:** Frequency of clinic menu requested from unique interactions.

Frequency	Unique visitors (N=409,768), n (%)
0	386,532 (94.33)
1	21,292 (5.20)
2	1635 (0.40)
3	246 (0.06)
≥4	63 (0.02)

**Table 5:** Activation and active use among unique users.

Activation and active use			Year								Total (N=409,768)
			2013 (N=43,746)		2014 (N=116,654)		2015 (N=151,903)		2016 (N=97,465)		
Activation, n (%)			11,470 (26.22)		34,830 (29.86)		77,839 (51.24)		64,765 (66.45)		188,904 (46.10)
Seek menu content, n (%)			11,246 (25.71)		33,551 (29.76)		67,725 (44.58)		56,696 (58.17)		169,218 (41.30)
Active use rate, n (%)			11,246 (98.05)		33,551 (96.33)		67,725 (87.01)		56,696 (87.54)		169,218 (89.58)
Keywords per month, mean (SD)			7.52 (6.09)		7.14 (5.96)		4.92 (6.23)		5.13 (6.49)		5.56 (6.34)
**Menu engagement, n (%)**											
	About family planning	N/A^a^		N/A		36,067 (23.74)		32,380 (33.22)		68,447 (16.70)	
	Family planning methods	30,492 (69.70)		79,438 (68.10)		70,379 (46.33)		36,113 (37.05)		216,422 (52.82)	
	Youth	N/A		N/A		24,317 (16.01)		21,920 (22.49)		46,237 (11.28)	
	Role model stories	10,185 (23.28)		27,580 (23.64)		17,438 (11.48)		12,674 (13.00)		67,877 (16.56)	
	Clinic	402 (0.92)		8,835 (7.57)		7,474 (4.92)		6,525 (6.69)		23,236 (5.67)	

^a^N/A=not applicable (“About family planning” and “Youth” menus not implemented until 2015).

**Table 6:** Information requested on different pregnancy prevention methods among unique users.

Pregnancy prevention methods, n (%)	Year				Total (N=409,768)
	2013 (N=43,746)	2014 (N=116,654)	2015 (N=151,903)	2016 (N=97,465)	
Natural family planning	13,451 (30.75)	33,827 (29.00)	33,126 (21.81)	18,565 (19.05)	98,969 (24.15)
Condom	9,332 (21.33)	24,807 (21.27)	21,263 (14.00)	10,586 (10.86)	65,988 (16.10)
Lactational amenorrhea method	8,263 (18.89)	22,009 (18.87)	23,258 (15.31)	13,163 (13.51)	66,693 (16.28)
Emergency contraception	8,655 (19.78)	21,778 (18.67)	18,264 (12.02)	9,167 (9.41)	57,864 (14.12)
Permanent	8,270 (18.90)	21,909 (18.78)	16,952 (11.16)	8,009 (8.22)	55,140 (13.46)
Implant	7,462 (17.06)	20,858 (17.88)	17,336 (11.41)	8,680 (8.91)	54,336 (13.26)
Injectable	6,786 (15.51)	18,903 (16.20)	17,050 (11.22)	8,593 (8.82)	51,332 (12.53)
Pills	5,865 (13.41)	16,208 (13.89)	13,183 (8.68)	6,344 (6.51)	41,600 (10.15)
Intrauterine contraceptive device	5,027 (11.49)	13,633 (11.69)	12,001 (7.90)	6,307 (6.47)	36,968 (9.02)

**Table 7:** Percent distribution of information requested on youth content among unique users.

Percent distribution, n (%)	Year		Total (N=249,368)
	2015 (N=151,903)	2016 (N=97,465)	
Puberty	8,366 (5.51)	7,677 (7.88)	16,043 (6.43)
About sex	18,144 (11.94)	16,398 (16.82)	34,542 (13.85)
Choices	6,756 (4.45)	6,482 (6.65)	13,238 (5.31)

### Loyalty

The active use rate was highest during the first 2 years of the program, with nearly all users (11,246/11,470, 98.05%, and 33,551/34,830, 96.33%, respectively) who accessed m4RH continuing on to query more specific content from the system. An explanation for the decrease in conversion rates may be a result of the timing and frequency of promotions, for example, radio and magazine advertisements (Table 8).

Table 9 shows respondent retention and loyalty. Overall, slightly more than one-third (150,915/409,768) of all new users accessing m4RH sent queries at least once per month for 2 or more months and 67.86% (278,088/409,768) of new and return users requested information multiple times per month.

**Table 8:** Promotion periods per year and interactions among new and return users.

Promotion periods and interactions		Year				Total
		2013	2014	2015	2016	
Promotional months per year, n		4	10	1	0	15
**Percent interactions, n (%)**						
	New users (N=409,768)	43,746 (100)	99,012 (84.88)	32,746 (21.56)	N/A^a^	175,504 (42.83)
	Return users (N=150,915)	4,731 (100)	31,084 (84.27)	6,877 (9.73)	N/A	42,692 (28.29)

^a^N/A=not applicable (No promotional periods during 2016).

**Table 9:** Percentage of return and repeat users per year.

Rates, n (%)	Year				Total (N=409,768)
	2013 (N=43,746)	2014 (N=116,654)	2015 (N=151,903)	2016 (N=97,465)	
Return users	4,731 (10.81)	36,887 (31.62)	70,703 (46.54)	38,594 (39.60)	150,915 (36.83)
Repeat users	30,685 (70.14)	80,542 (69.04)	100,749 (67.64)	64,112 (65.78)	278,088 (67.86)
Conversion rate	11,470 (25.71)	33,551 (28.76)	67,725 (44.58)	56,696 (58.17)	169,218 (41.30)

**Figure 3:**
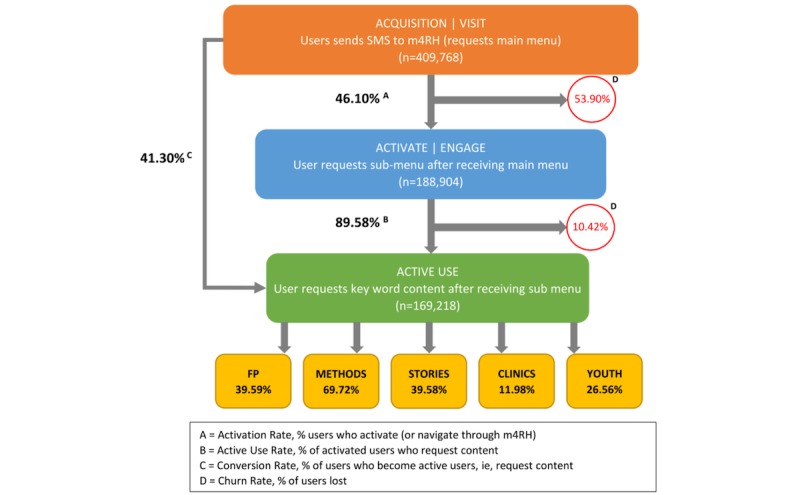
Mobile for Reproductive Health respondent progression. m4RH: Mobile for Reproductive Health; SMS: short message service.

On average, users accessed the system for 3 months over the 4-year period. This is also demonstrated by the average number of keywords queried per month presented in sections “Activate” and “Active Use” (Figure 3). Of the 409,768 new users acquired from September 2013 to October 2016, 41.30% (169,218/409,768) became active users seeking further content (Figure 3). At the end of the study period, there were a total of 169,218 active users (users who requested specific keyword content). Among these users, the majority (117,805/169,218, 69.62%) of them requested content on pregnancy prevention methods, and only 11.98% (20,277/169,218) sought information on FP clinics.

Promotional periods were present for 15/36 months during the study period. However, as shown in Table 8, nearly all promotional activities took place in 2013 and 2014, and no promotional activities were reported for 2016. Looking specifically at 2014, 84.88% (99,012/116,654) of new users and 84.27% (31,084/36,887) of return users were acquired during months when promotional activities were being conducted. The unequal distribution of promotional activities makes it difficult to establish the influence of promotional activities on user engagement.

## Discussion

### Principal Findings

Our study shows that approximately 67.86% (278,088/409,768) of users accessed the system more than once within a month period, averaging 5.56 times a month. Since m4RH is an on-demand system requiring people to access the service voluntarily, our results may suggest that the users have a keen interest in information to enhance their contraceptive knowledge. Our results also suggest a high degree of demand and motivation among users who access the service. A conversion rate of 41.30% (169,218/409,768) was observed among all users who accessed m4RH in a 3-year period, and an active use rate of 89.58% (169,218/188,904) among all who chose to navigate through the service menu.

More users were lost in the first step than in the second step of the navigation menu, the churn rates being 53.90% (220,864/409,768) and 10.42% (19,686/188,904), respectively. The churn rate could be explained by several factors. First, the fact that most users drop during the first stage of the navigation may be reflective of curious users who are trying out the service but decide they are not interested in going further. Second, it could suggest problems with message delivery (technology challenges have been observed contributing to delayed, truncated, or lack of message delivery). Third, although m4RH was developed with considerable formative work that allowed end users to contribute to the design of the service [18], this dropoff could raise the question of whether our “pull” design across all stages, with its multiple navigation steps structure, discourages users from accessing content. Alternatively, the dropoff could signify that menu option descriptions are unclear to users. Despite these potential challenges, we observed a high level of repeat use across all years. Close attention to churn rate data could help the program implement measures to reduce user attrition to further increase active use. For example, a cluster-randomized trial using hybrid text messaging and face-to-face interventions to reduce teenage pregnancies in Denver, Colorado, USA, was able to reduce user drop-out rates through the use of follow-ups by the program facilitators [19]. Analysis of churn rate data can allow program managers to identify the need to implement innovative solutions such as those applied by the Denver team. Because few programs have reported their activation rate, we are unable to determine how our findings compare to what is observed in similar programs.

Our study examined user engagement with service promotion. While we were unable to determine a statistically significant relationship between promotion and increased use, we did observe the greatest number of new users during the months when promotion activities occurred. Our findings show that during 2014, 84.88% (99,012/116,654) of new users and 84.27% (31,084/36,887) of return users were acquired during months when promotional activities were being conducted. This finding supports the rationale for investing in mass media promotion to influence m4RH use. Information about patterns in the time of day when users access the system can provide important data for future promotional efforts. Peak times for users to access m4RH across all 4 years were 1 pm and 8 pm. This finding helps to inform the timing of radio promotions to get maximum responses, as studies have shown that people are likely to respond to cues to action soon after they receive them [20].

Behavioral experts recommend that health messages should be tailored to audiences so that they can be personally relevant and affect behavioral change [21]. Past surveys to assess the demographic profile of users repeatedly showed that m4RH is mostly utilized by young people. This finding influenced the addition of youth content in 2015 as a means to tailor messages to this audience segment. However, only 11.28% (46,237/409,768) of all users and 26.56% (44,943/169,218) of active users requested youth content. This could suggest a higher demand for specific contraceptive information compared with general adolescent and youth SRH information that is also offered through other communication channels, for example, through HIV or AIDS health promotion programs. However, because the m4RH system did not routinely collect demographic data from users, we are unable to disaggregate information-seeking behavior by age or sex. Our results show that m4RH users mostly requested content on contraceptive methods and role model stories. Relatively few users requested information about clinic locations, suggesting that this content may not be interesting to most users of the m4RH system or users may have been discouraged by the need to enter additional information (first 3 or 4 letters of their ward). It is notable that the 4 regions with the most requests were all urban areas. This may be a result of greater promotional activity or higher levels of mobile phone ownership in urban areas [22]. However, this finding may be skewed as we know certain populations (key populations and youth) will travel over long distances to visit clinics in urban settings to protect their confidentiality. The request for clinic information from these locations may reflect this trend.

Analysis of data about information-seeking behavior has important implications for future iterations of the m4RH program and other health promotion programs with similar target audiences and information-sharing goals.

### Challenges and Limitations

The m4RH system design presented some challenges in understanding respondent progression through the menus. As described above, the system was designed so that a user first queried information from the main menu and then from submenus. However, it appears that over time, respondents would become familiar with numerical codes assigned to menu items and directly access content without first accessing the main menu or submenus. Initially, we intended to identify repeat users by summing the total number of unique phone numbers that sent “m4RH” more than once. However, we realized that this definition of repeat use did not account for users who re-entered the system without first SMS text messaging “m4RH.” This required us to create a more sophisticated Stata code that instead summed the total number of unique phone numbers that sent any code to the system on >1 day. Respondent queries (either initial or follow-up), including invalid numerical codes, text, or a combination of both, also presented challenges for analysis. For example, a respondent interested in pregnancy prevention methods (menu code 66) might send a message reading “m4RH 66,” and although this query would be received by the m4RH system, the respondent would not receive any information on pregnancy prevention methods.

Information on use patterns alone does not paint a full picture of engagement [10]. Previously conducted and published research on the m4RH program in Tanzania and elsewhere sheds light on the program’s feasibility and acceptability [18,23]. However, the m4RH program in Tanzania did not routinely collect qualitative and demographic data, information that would have helped us understand the nature of engagement. For example, questions about why users drop off the navigation menu before they access content or whether there are variations in information preferences across age and gender groups would inform further design improvements.

### Conclusions

The vertical and horizontal scale-up and wide reach of m4RH in Tanzania has been a major success story [24,25]. The rich data captured over the 3-year timeframe of this analysis and the results of our analyses provide insights into a useful framework to measure the degree and nature of user engagement using routine system data. For example, the conversion rate suggests a need to identify approaches to improve user engagement beyond their first contact with the m4RH system, and our data on the clinic locater use suggests that it may not provide value in its current form. Additional data collection efforts could provide a deeper understanding of our findings. These analyses contribute to knowledge about how users engage with SMS text messaging-based health promotion interventions and demonstrate how data generated on user interactions could inform improvements to the design and delivery of a service, thereby enhancing its effectiveness.

## Abbreviations

FP: family planning
IUCD: intrauterine contraceptive device
m4RH: Mobile for Reproductive Health
mHealth: mobile health
SMS: short message service
SRH: sexual and reproductive health
STI: sexually transmitted infection

## References

[1] High-Impact Practices in Family Planning (HIP) (2013). mHealth: Mobile technology to strengthen family planning programs.

[2] Hall AK, Cole-Lewis H, Bernhardt JM (2015). Mobile text messaging for health: a systematic review of reviews. Annu Rev Public Health.

[3] Orr JA, King RJ (2015). Mobile phone SMS messages can enhance healthy behaviour: a meta-analysis of randomised controlled trials. Health Psychol Rev.

[4] Irvine L, Falconer DW, Jones C, Ricketts IW, Williams B, Crombie IK (2012). Can text messages reach the parts other process measures cannot reach: an evaluation of a behavior change intervention delivered by mobile phone?. PLoS One.

[5] Yardley L, Spring BJ, Riper H, Morrison LG, Crane DH, Curtis K, Merchant GC, Naughton F, Blandford A (2016). Understanding and Promoting Effective Engagement With Digital Behavior Change Interventions. Am J Prev Med.

[6] Perski O, Blandford A, West R, Michie S (2016). Conceptualising engagement with digital behaviour change interventions: a systematic review using principles from critical interpretive synthesis. Transl Behav Med.

[7] Alkhaldi G, Modrow K, Hamilton F, Pal K, Ross J, Murray E (2017). Promoting Engagement With a Digital Health Intervention (HeLP-Diabetes) Using Email and Text Message Prompts: Mixed-Methods Study. Interact J Med Res.

[8] Couper MP, Alexander GL, Zhang N, Little RJA, Maddy N, Nowak MA, McClure JB, Calvi JJ, Rolnick SJ, Stopponi MA, Cole JC (2010). Engagement and retention: measuring breadth and depth of participant use of an online intervention. J Med Internet Res.

[9] Johnson Douglas, Juras Randall, Riley Pamela, Chatterji Minki, Sloane Phoebe, Choi Soon Kyu, Johns Ben (2017). A randomized controlled trial of the impact of a family planning mHealth service on knowledge and use of contraception. Contraception.

[10] Smith C, Vannak U, Sokhey L, Ngo TD, Gold J, Free C (2016). Mobile Technology for Improved Family Planning (MOTIF): the development of a mobile phone-based (mHealth) intervention to support post-abortion family planning (PAFP) in Cambodia. Reprod Health.

[11] McClure D (2007). Startup Metrics for Pirates: AARRR !!! (Startup Metrics for Product Marketing & Product Management).

[12] Vahdat HL, L'Engle KL, Plourde KF, Magaria L, Olawo A (2013). There are some questions you may not ask in a clinic: providing contraception information to young people in Kenya using SMS. Int J Gynaecol Obstet.

[13] L'Engle KL, Vahdat HL, Ndakidemi E, Lasway C, Zan T (2013). Evaluating feasibility, reach and potential impact of a text message family planning information service in Tanzania. Contraception.

[14] Ritterband LM, Thorndike FP, Cox DJ, Kovatchev BP, Gonder-Frederick LA (2009). A behavior change model for internet interventions. Ann Behav Med.

[15] Short CE, Rebar AL, Plotnikoff RC, Vandelanotte C (2015). Designing engaging online behaviour change interventions: A proposed model of user engagement. The European Health Psychologist.

[16] Taki S, Lymer S, Russell CG, Campbell K, Laws R, Ong K, Elliott R, Denney-Wilson E (2017). Assessing User Engagement of an mHealth Intervention: Development and Implementation of the Growing Healthy App Engagement Index. JMIR Mhealth Uhealth.

[17] Ministry of Health, Community Development, Gender, Elderly and Children (MOHCDGEC) [Tanzania Mainland], Ministry of Health (MOH) [Zanzibar], National Bureau of Statistics (NBS), Office of the Chief Government Statistician (OCGS), and ICF (2016). Tanzania Demographic and Health Survey and Malaria Indicator Survey (TDHS-MIS) 2015-16.

[18] Van Rossem R, Meekers D (2007). The reach and impact of social marketing and reproductive health communication campaigns in Zambia. BMC Public Health.

[19] Bull SS, Levine DK, Black SR, Schmiege SJ, Santelli J (2012). Social media-delivered sexual health intervention: a cluster randomized controlled trial. Am J Prev Med.

[20] Devine S, Leeds C, Shlay JC, Leytem A, Beum R, Bull S (2015). Methods to assess youth engagement in a text messaging supplement to an effective teen pregnancy program. J Biomed Inform.

[21] L'Engle KL, Vahdat HL, Ndakidemi E, Lasway C, Zan T (2013). Evaluating feasibility, reach and potential impact of a text message family planning information service in Tanzania. Contraception.

[22] (2016). Tanzania Telecommunications Report Q4 2016.

[23] High-Impact Practices in Family Planning (HIP) (2012). Health communication: enabling voluntary and informed decision-making.

[24] World Health Organization (2017). WHO Strategic Communications Framework for Effective Communications.

[25] L'Engle K, Plourde KF, Zan T (2017). Evidence-based adaptation and scale-up of a mobile phone health information service. Mhealth.

